# Gender and ethnic diversity in randomised clinical trials in age-related macular degeneration and diabetic macular oedema

**DOI:** 10.1038/s41433-025-03595-7

**Published:** 2025-02-20

**Authors:** Farah NI Ibrahim, Sobha Sivaprasad, Chui Ming Gemmy Cheung

**Affiliations:** 1 Singapore National Eye Centre, Singapore, Singapore; 2 National Institute of Health Research Moorfields Biomedical Research Centre, Moorfields Eye Hospital NHS Foundation Trust, London, UK; 3 Duke-NUS Medical School, National University of Singapore, Singapore, Singapore

**Keywords:** Health care, Epidemiology

## Abstract

In recent years, there has been increasing recognition of the importance of diversity in pivotal randomised clinical trials (RCTs). This is vital to ensure the validity and applicability of the results in the clinical setting. In this review, we aim to assess the inclusion of females and minoritized groups in recent RCTs in age-related macular degeneration (AMD) and diabetic macular oedema (DMO) and explore any potential barriers to their enrolment. Overall, a female predominance was observed among the AMD RCTs while less than half of the study population in DMO trials were females. White participants made up the majority of the study population in both AMD and DMO trials. Gender distribution within minoritized groups has only been reported in a few trials but appears lower than in the white population. This disparity may be attributable to the difference in the prevalence of diseases between these subgroups, as well as social and/ or cultural reasons. Nonetheless, there has been an overall increase in representation of minoritized groups over the past two decades. These observations provide important perspectives to consider when applying clinical trial learnings to clinical settings.

## Introduction

Results from randomised clinical trials (RCTs) provide a high level of evidence for evaluating novel therapies and are important in guiding the treatment of major diseases in ophthalmology. It is therefore vital that RCTs represent real-world populations, as poor representation has an impact on the generalisability of the results. In an increasingly diverse population, racial and ethnic differences in eye disease prevalence and course of progression have been reported in various epidemiological studies [1]. In recent years, there has been growing recognition and efforts to increase the participation of females and racial/ ethnic minoritized groups; namely those distinguished by physical or cultural traits, who face unequal and different treatment within their society. In the United States, this includes Black/African Americans, Hispanics, Asian Americans, Native Americans and Pacific Islanders [2]. For example, the National Institutes of Health (NIH) Revitalization Act mandated the inclusion of minoritized groups in NIH-funded studies in 1993 [3]. Following that, the US Food and Drug Administration (FDA) implemented guidelines encouraging greater participation of females, and the confirmation of the Final Rule in 2017 expanded the requirements for reporting race and ethnicity data on clinical trial participants [4].

The objective of this review is to evaluate the representation of minoritized groups in pivotal age-related macular degeneration (AMD) and diabetic macular oedema (DMO) RCTs and explore any potential barriers to their enrolment.

## Methods

Institutional review board approval was not required for this study as no protected patient information was accessed. We reviewed the registration trials for ranibizumab, aflibercept, brolucizumab, faricimab, aflibercept high dose (8 mg), pegcetacoplan and avacincaptad pegol from 2006 to 2023, and analysed the proportion of participants according to gender and ethnicity.

## Results

Twenty-two RCTs over the 17-year period were included [9 in neovascular AMD (nAMD), 9 in DMO, and 4 in geographic atrophy (GA) secondary to AMD] (Table 1). Overall, the majority of participants were White. Female participants were more in nAMD and GA studies, while male participants were more represented in DMO studies. However, gender within minoritized groups has not been reported, apart from a few minor exceptions.

**Table 1:** Registration trials in exudative age-related macular degeneration (nAMD), diabetic macular oedema (DMO) and geographic atrophy (GA) from 2006 to 2023.

Condition	Study	Year	Female (%)	White (%)	Hispanic	Black	Asian (%)	Female proportion in Asian subgroup (%)	Other
					(%)	(%)			
AMD	MARINA	2006	64.8	96.7	NA*	NA*	NA*	NA*	3.3
	ANCHOR	2006	55.2	97.9	NA*	NA*	NA*	NA*	2.1
	VIEW 1	2012	58.8	96.6	NA*	0.24	0.1	29.4	2.14
	VIEW 2	2012	55.5	72.3	NA*	0.4	21.3		5.49
	HAWK	2019	56.5	81.1	NA*	0.36	14.7	NA*	3.98
	HARRIER	2019	57.1	92.2	NA*	0.26	6.1	NA*	1.62
	TENAYA	2022	60	90	7.74	0.44	9	28.3	0.44
	LUCERNE	2022	59	83.3	12.31	1.06	10.9		0.15
	PULSAR	2023	54.5	75.8	2.8	0.4	23.2	NA*	NA*
DMO	RIDE**	2012	41.9	81.7	26.4	10.9	3.1	NA*	4.54
	RISE**	2012	44.5	77.2	18.3	13.5	5.2	NA*	2.91
	VIVID	2014	37.9	79	NA*	0.49	19.5	6.4 [*Japan*]	0.25
	VISTA	2014	44.8	82.4	NA*	10.9	2.1	NA*	3
	KITE	2022	34.7	73.6	NA*	1.11	25.3	NA*	20.8
	KESTREL	2022	37.3	81.6	NA*	4.24	13.6	NA*	13.95
	YOSEMITE	2022	40.2	78	12.12	6.27	8.9	42.3	2.44
	RHINE	2022	39.1	79	21.13	6.83	10.7	NA*	0.31
	PHOTON	2023	39	72	18	9	15	NA*	2
GA	OAKS	2023	61.2	91.5	NA*	NA*	NA*	NA*	NA*
	DERBY	2023	60.5	94.0	NA*	NA*	NA*	NA*	NA*
	GATHER 1	2023	70.1	98.3	NA*	NA*	NA*	NA*	NA*
	GATHER 2	2023	69.4	82.3	NA*	NA*	NA*	NA*	NA*

*AMD* age-related macular degeneration, *DMO* diabetic macular oedema, *GA* geographic atrophy.

NA*- information not reported.

**Patients of more than 1 race were counted for each category they indicated.

### Gender distribution

Overall, a female predominance was observed among the nAMD RCTs. As one of the earliest trials evaluating ranibizumab in AMD, MARINA enrolled participants from 96 sites in the United States [5] and ANCHOR from 83 international sites [6]. The proportion of female participants was 64.8% (MARINA) and 55.2% (ANCHOR). In subsequent studies (VIEW 1, VIEW 2, HAWK, HARRIER, TENAYA, LUCERNE, PULSAR), female participants made up 55–60% of the study population [7–10].

In the DMO trials, females made up less than half of the study population. RISE and RIDE (2012) evaluated ranibizumab and enrolled 377 and 382 participants from the United States and South America [11]. Females accounted for 41.9% and 44.5% of all participants. In subsequent studies, (VIVID, VISTA, KITE, KESTREL, YOSEMITE, RHINE, PHOTON), female participants made up 34.7%–44.8% of the study population. [10, 12–14]

In the latest studies which evaluated GA, female participants accounted for 60.5%–70.1% [15–17].

As AMD is more prevalent in females [18, 19] and DMO in males [20], these trials represent the real-world male: female proportions.

### Ethnic representation

For both nAMD and DMO, White participants made up the vast majority of the study population. In MARINA and ANCHOR, White participants made up 96.7% and 97.9% of the study population, respectively [5, 6]. The representation of minoritized groups was classified as ‘Other’ and no further sub-classification was reported. Later trials have described other racial groups more specifically. VIEW 1 and VIEW 2 (2012), as well as HAWK and HARRIER (2019), subcategorised other racial groups into Blacks and Asians. VIEW 1 was conducted in North America while VIEW 2 included European and Asian sites. The proportion of White participants was 96.6% in VIEW 1 and 72.3% in VIEW 2 [7]. The proportion of Asian and Black participants was 0.1% and 0.24% in VIEW 1, 21.3% and 0.4% in VIEW 2 respectively. HAWK and HARRIER (2019) were conducted in 408 sites in the United States (HAWK) and Europe, Asia, South Australia and Japan (HARRIER) [8]. While White participants were still the majority (81.1% in HAWK and 92.2% in HARRIER), 14.7% and 6.1% Asians were included in these studies. Black participants were also included (0.36% in HAWK, 0.26% in HARRIER). In TENAYA and LUCERNE (2022), Whites accounted for 90% and 83.3% of all participants [9]. Only 9% and 10.9% were of Asian descent, with 0.44% and 1.06% of Black participants. These were also some of the first clinical trials in AMD which reported Hispanic representation (7.74% in TENAYA and 12.31% in LUCERNE). The latest RCT for AMD was PULSAR (2023) which included a majority of Whites (75.8%) and one of the highest proportion of Asian participants (23.2%) [10].

In the DMO trials, RISE and RIDE (2012) included 81.7% and 77.2% White participants, respectively [11]. There were 3.1% and 5.2% Asian participants enrolled. Hispanic participants accounted for 26.4% and 18.3%, while Black participants accounted for 10.9% and 13.5% in these trials. The VIVID and VISTA studies also included a majority of White participants (79% and 82.4%). 0.49% and 10.9% of participants were Black, while Asians accounted for 19.5% and 2.1% of all participants respectively [12]. In KITE and KESTREL (2022), White participants accounted for the majority of participants (73.6% and 81.6%), while the proportion of Asians was 25.3% and 13.6% [13]. These studies had one of the highest proportions of Asian representation among all the other RCTs. Black participants were also included (1.11% and 4.24%), while no Hispanic representation was reported. YOSEMITE and RHINE (2022) included 78% and 79% participants categorised as White [14]. Asians accounted for 8.9% and 10.7% of all participants. There was a significant proportion of Hispanic representation included (12.12% and 21.13%). PHOTON (2023) included a majority of Whites (72%) and Asians accounting for 15% of all participants [21].

In the registration studies for the 2 new treatments for GA secondary to AMD, 82.3–98.3% were Caucasians [15–17].

### Female gender among minoritized groups

Although data on the proportion of female participants from minoritized groups would have been captured, few studies have reported these data. A post-hoc analysis of the Asian subpopulation of the VIEW trials demonstrated that female participants represented only 29.4% in this subgroup [22]. In a pooled 1-year outcome analysis of TENAYA/LUCERNE trials in Asian participants, the proportion of female representation in this subgroup was 28.3% [23]. In the PULSAR study Asian subgroup analysis, 30% of the Asian subgroup were females.

Among DMO trials that have reported the gender make up of ethnic subgroups, an analysis of Japanese participants in the VIVID study revealed the proportion of female participants was a mere 6.4% [24]. A subgroup analysis of Asian participants in YOSEMITE and RHINE reported that 42.3% were females, one of the highest representation reported [25].

## Discussion

The prevalence of AMD in the United States is approximately 12.6% (19.8 million people), with different rates reported across ethnic groups [18]. White individuals have the highest prevalence (5.4%), followed by Chinese (4.6%), Hispanics (4.2%) and Blacks (2.4%) [26]. Some findings also suggest higher rates of AMD in females than males, particularly in older age groups. The highest prevalence of any AMD is reported in those 75–84 years old, varying from 7.4% in Blacks to 15.8% in Whites and Chinese. In DMO, males exhibit higher prevalence compared to females, with an odds ratio of 1.81 for DMO occurrence. Significant variations were observed across ethnicities, with a reported prevalence of DMO ranging from 4.2% to 12.8% in Asian populations, depending on the severity and duration of diabetes [27].

In an increasingly diverse population, adequate representation of minoritized groups helps ensure that the results of RCT can be generalisable to the clinical setting. This is vital in diseases with significant differences in prevalence, natural history and treatment response. For example, the VIVID and VISTA studies had similar proportions of patients with baseline Diabetic Retinopathy Severity Scale (DRSS) scores of low risk (≤43), moderate risk (47), and high risk (≥53): 44% versus 24.5%, 18.3% versus 17.1% and 36.8% versus 32% [12]. In contrast, the VIVID-EAST study included a significantly higher proportion of Asian patients with high-risk DRSS scores (15.9% low risk, 18.5% moderate risk and 65.3% high risk) [28]. Similarly, polypoidal choroidal vasculopathy (PCV) is a subtype of nAMD which is more common among Asians and may respond differently to treatment options [29]. Another important reason is that different subgroups may demonstrate different safety profiles for the same treatment. For example, females were reported to be at higher risk for brolucizumab-related intraocular inflammation (IOI) [30]. Also, higher rates of brolucizumab-related IOI have been reported in Japanese patients [31].

Over the past two decades, there has been an overall increase in Asian and Black subgroups recruited into the registration trials, but the vast majority of participants remain predominantly White. In some of the newer trials, there also has been an increase in reporting representation from the Hispanic community. To address the under-representation, follow-up trials targeting specific subgroups have also been conducted, such as the VIVID-EAST study evaluating DMO in Asian participants, the PLANET and SALWEEN studies which evaluate treatment response in PCV, and the ELEVATUM study evaluating Faricimab in DMO in under-represented ethnic minority subgroups in the United States. With the advent of new treatment options for geographic atrophy, further studies with representation from various ethnic groups may be worthwhile, as significant differences in GA phenotypes and growth rates have been reported in Asian patients compared to non-Asians [32].

A new observation we wish to highlight is that a notably lower proportion of females were observed within minoritized groups. This may be attributable to several possible reasons. First, there may be a significant difference in the prevalence of diseases between these subgroups. For example, while nAMD has been reported to be more common in females in the Western population, the PCV subtype, which is more common among Asians, has been reported to be more prevalent in males [33]. In contrast, such disparity has not been reported for DMO [34]. Given the higher prevalence of AMD among women, we found an overall greater percentage of female participants in AMD studies. This can be seen as an appropriate reflection of the demographic reality of the disease. Conversely, male participation in DMO trials tends to be greater, aligning with that complications from diabetes are more common in the male population [20].

Another possible explanation for the disparity observed is inadequate demographic reporting in the earlier RCTs. However, Yu et al. reported an increased trend of racial reporting in DMO clinical trials between 2002 and 2021 [35]. They also observed that the enrolment of Hispanic and Asian patients has increased with a subsequent decrease in the enrolment of White participants across the 2 decades. It is also worth noting that heterogeneity also exists among those identified as Whites. In RISE and RIDE, patients of more than 1 race were counted for each category they indicated. There is a broader need for this awareness and inclusion in our increasingly diverse population, to be considered for future RCTs.

A likely association of the under-representation of minoritized groups lies in the socioeconomic aspects. In developing countries in Asia, poverty and income constraints contribute to problems accessing healthcare. Additional constraints include lower rates of health insurance coverage, less education and lack of economic independence [36]. The socioeconomic position of minoritized groups is intricately intertwined with behavioural aspects which in turn can influence their financial status. It is therefore challenging to attribute socioeconomic status based on gender and ethnicity alone. Participation in RCTs requires additional costs such as additional transport and more frequent and prolonged clinical visits. Operational efforts to break down these barriers can include providing patient compensation and stipend, providing fellow eye treatment and providing patient transportation to and from study visits. This could be a potential incentive for patients from lower socioeconomic status to participate in RCTs, in order to receive treatment which, they would have otherwise not been able to afford.

A significant proportion of the study population in AREDS (32%) and AREDS2 (45%) included those with an educational level of at least a bachelor’s degree, with the differences attributed to the older population of the AREDS2 cohort [37]. This suggests that patients from a certain level of socioeconomic status tend to be recruited into RCTs. Educated patients are also able to comprehend complex study designs and risks, and having a better understanding may encourage them to participate. A review by Nakayama et al. reported that most of the ophthalmological RCTs are from populations of high-income and upper-middle-income countries, with only 0.25% from low-income countries where the burden of disease is actually highest [38]. This highlights the importance of outreach efforts through community-based organisations in recruiting clinical trial participants, particularly for difficult-to-reach populations and those with limited health literacy.

Finally, cultural traditions and social roles may also be a factor. Asian women may be less willing to discuss clinical trials as they often rely on their carers for help with their decisions. This is in contrast with a study of African-American women, whose decisions do not rely on other individuals [39]. Family involvement plays a significant role in individual patient autonomy and decision-making in the Asian culture, in contrast with American norms [40]. This emphasises the importance of handling recruitment and consent procedures in RCTs with greater cultural competence, for example, by addressing language barriers. English proficiency is usually required for trial participation. Offering consent forms and supplementary materials about RCTs to patients in languages other than English can encourage participation in addition to allowing patients to make informed decisions. Hiring multilingual staff and engaging clinicians who primarily serve particular communities can foster trust in the medical research community and increase participation among minoritized groups.

Our findings can have implications not only for clinical practice but also for future RCT design. Clinicians should be aware that the results of RCTs may not be fully applicable in clinical settings, especially when treating certain populations. This is particularly important when using new drugs or treatment options, as well as counselling patients on expected outcomes. Subsequent RCTs should consider the actual demographic diversity of the disease, making efforts to include a population with very specific inclusion and exclusion criteria that ensures good compliance with both the treatments as well as the follow-up, in order to decrease the generalisability of a broadly diverse population. Epidemiological data should be considered as a more suitable reference for trial enrolment rather than solely census data [41]. Incorporating strategic diversity planning early in trial design helps ensure adequate representation from the outset, and other characteristics such as age, comorbidities and socioeconomic status should be considered in addition to balancing gender and ethnicity.

In efforts to increase gender and ethnic diversity in RCTs, reducing obstacles to participation for under-represented groups should be a priority. Using the strategies discussed above, concerted efforts to provide support to minoritized groups should be prioritised and sustained, to ensure equitable access to resources, opportunities and representation.

In conclusion, while there have been increased efforts to include non-White participants in major RCTs, the proportion of Asians is limited. Females accounted for roughly half of the overall study population in RCTs, but appear to be notably under-represented among ethnic minority subgroups. These observations provide important perspectives to consider when applying clinical trial learnings to clinical settings. Equal gender representation among ethnic racial groups may need further attention in the future.
